# Exome Sequencing Is an Efficient Tool for Variant Late-Infantile Neuronal Ceroid Lipofuscinosis Molecular Diagnosis

**DOI:** 10.1371/journal.pone.0109576

**Published:** 2014-10-15

**Authors:** Liliana Catherine Patiño, Rajani Battu, Oscar Ortega-Recalde, Jeyabalan Nallathambi, Venkata Ramana Anandula, Umashankar Renukaradhya, Paul Laissue

**Affiliations:** 1 Unidad de Genética, Grupo GENIUROS, Escuela de Medicina y Ciencias de la Salud, Universidad del Rosario, Bogotá, Colombia; 2 Department of Vitreoretina, Narayana Nethralaya, Bangalore, India; 3 GROW Research Laboratory, Narayana Nethralaya, Narayana Health City, Bangalore, India; 4 Department of Molecular Diagnostics, Narayana Nethralaya, Bangalore, India; University of Sydney, Australia

## Abstract

The neuronal ceroid-lipofuscinoses (NCL) is a group of neurodegenerative disorders characterized by epilepsy, visual failure, progressive mental and motor deterioration, myoclonus, dementia and reduced life expectancy. Classically, NCL-affected individuals have been classified into six categories, which have been mainly defined regarding the clinical onset of symptoms. However, some patients cannot be easily included in a specific group because of significant variation in the age of onset and disease progression. Molecular genetics has emerged in recent years as a useful tool for enhancing NCL subtype classification. Fourteen NCL genetic forms (CLN1 to CLN14) have been described to date. The variant late-infantile form of the disease has been linked to *CLN5*, *CLN6*, *CLN7* (*MFSD8*) and *CLN8* mutations. Despite advances in the diagnosis of neurodegenerative disorders mutations in these genes may cause similar phenotypes, which rends difficult accurate candidate gene selection for direct sequencing. Three siblings who were affected by variant late-infantile NCL are reported in the present study. We used whole-exome sequencing, direct sequencing and *in silico* approaches to identify the molecular basis of the disease. We identified the novel c.1219T>C (p.Trp407Arg) and c.1361T>C (p.Met454Thr) *MFSD8* pathogenic mutations. Our results highlighted next generation sequencing as a novel and powerful methodological approach for the rapid determination of the molecular diagnosis of NCL. They also provide information regarding the phenotypic and molecular spectrum of CLN7 disease.

## Introduction

The neuronal ceroid-lipofuscinoses (NCL), also known as Batten Disease, is a group of neurodegenerative disorders which is characterised by the accumulation of autofluorescent storage positive material in the cytoplasm of neurons [1], [2]. This feature can also be found in other tissues, such as the skin and skeletal muscle [3]. NCL is one of the most frequently inherited childhood onset neurodegenerative pathologies since its prevalence ranges from 1∶100.000 to 1∶1.000.000 [2]. From a clinical point of view, NCL patients suffer from epilepsy, visual failure, progressive mental and motor deterioration, myoclonus, dementia and reduced life expectancy [1]. Classically, NCL-affected individuals have been classified into six categories (congenital, infantile, late infantile, variant late infantile, juvenile and adult) which have been mainly defined regarding the clinical onset of symptoms [4]. However, some patients cannot be easily included in a specific group because of significant variation in the age of onset and disease progression. Molecular genetics has emerged in recent years as a useful tool for enhancing NCL subtype classification. Fourteen NCL genetic forms (CLN1 to CLN14) have been described to date [2], [5]. More than 360 NCL aetiological mutations have been reported, most of which have been included in the NCL Mutation Database (http://www.ucl.ac.uk/ncl/mutation) [6].

To date, the variant late infantile form of the disease has been linked to *CLN5*, *CLN6*, *CLN7* and *CLN8* mutations [2], [7]. The *MFSD8* (*CLN7*) gene, which is located on chromosome 4q28.1–q28.2, encodes for the MFSD8 (*major facilitator superfamily domain-containing protein 8*) protein [8]. This factor belongs to the major facilitator superfamily (MFS) family of membrane transport proteins which are involved in transporting small solutes through cell membranes. It has been suggested that MFSD8 acts as a lysosomal transporter in eukaryotic cells. Although this protein is ubiquitously expressed, high transcript concentrations have been identified in specific brain locations, such as the cerebellar cortex and the hippocampus [9].

More than 30 pathogenic sequence variants have been described so far in *MFSD8*, most being homozygous missense mutations (www.ucl.ac.uk/ncl/mutation) [2]. It has been proposed that this kind of mutations does not affect protein subcellular location but might produce disturbances regarding their functional properties ([6] and references therein). Almost all *MFSD8* mutations are isolated and some founder effects have been described [10]. Despite advances in the diagnosis of neurodegenerative disorders, CLN7 disease remains clinically difficult to distinguish from other forms of late-infantile lipofuscinosis. Molecular testing thus constitutes an indispensable technique for establishing an accurate diagnosis and facilitating genetic counselling.

Three siblings who were affected by variant late-infantile NCL are reported in the present study. Whole-exome sequencing led to identify the novel c.1219T>C (p.Trp407Arg) and c.1361T>C (p.Met454Thr) *MFSD8* pathogenic mutations and to define the clinical and molecular diagnosis precisely. These results highlighted next generation sequencing as a powerful methodological approach for the rapid determination of the molecular basis of NCL. They also provide information regarding the phenotypic and molecular spectrum of CLN7 disease.

## Material and Methods

### Patients

**Figure 1:**
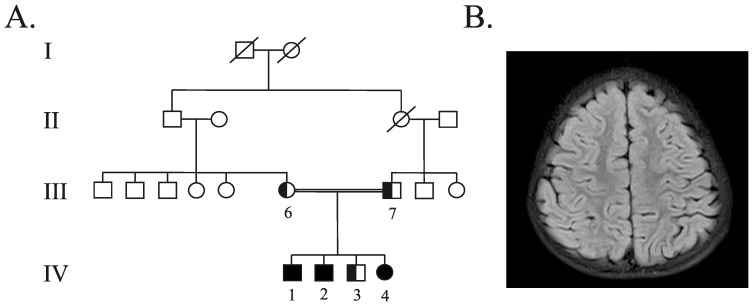
A) Pedigree of the NCL family. Black symbols refer to affected individuals. Half-black symbols into third and fourth generation individuals symbols (III:6, III:7, IV:3) represents the *MFSD8* c.1219T>C and c.1361T>C mutations at heterozygous state. **B)** Brain magnetic resonance imaging (T2-FLAIR image, axial section) of patient (P3) showing mild cerebral atrophy.

The patients (P1-IV:1, P2-IV:2 and P3-IV:4) and a non-affected son (C3-IV:3) were siblings who were attending Retina clinic at Narayana Nethralaya, (Bangalore, Karnataka, India), along with their parents (C1-III:6 and C2-III:7) (**Figure 1A**). This study was approved by the Universidad del Rosario's and Narayana Nethralaya eye hospital's Ethics Committees and was conducted in line with the Declaration of Helsinki. The parents gave their written informed consent and signed on behalf of their children. The patients' parents were reported to be consanguineous. All three patients (P1, P2 and P3) failed a school vision test at the age of 5–6 years and were referred for an eye check-up. All three children subsequently developed seizures and regression of motor milestones noted in the older two siblings.

P1 is a 14 year old male child seen at the age of 5 years with rapid decrease in vision in both eyes. Vision at presentation was 6/90 (Snellen's) in both eyes. Fundus examination showed severe optic disc pallor and arteriolar attenuation. A full field electroretinogram (ERG) showed severe rod-cone dysfunction. He developed seizures at the age of 7 years, and was noted to have progressive neurological illness in the form of recurrent myoclonic jerks and generalized tonic clonic seizures. There was a progressive cognitive decline and visual impairment. Last examination showed vision of perception of light (PL) in both eyes. Currently, he is completely bed-bound with very poor vision and uncontrolled seizures on multiple anticonvulsants. Electroneuromyogram done on the upper limbs showed bilateral CPN axonal neuropathy. MRI brain was not available for this patient.

P2 is a 12 year old male who was referred since he failed a school vision examination at the age of 6 years. He had useful vision at birth. Examination showed a visual acuity of 2/60 in both eyes. Fundus showed bilateral pale optic discs, severe arteriolar attenuation with diffuse pigment mottling throughout the fundus and few bony spicules. Spectral-domain optical coherence tomography (SD-OCT) showed diffuse loss of photoreceptors. Full field ERG showed severe attenuation of the rod and cone responses. He developed seizures at the age of 7 years with a regression of motor milestones. When examined last (at the age of 12 years), his vision was PL in both eyes. He had slurred speech with difficulty in walking and needed assistance. Neurological examination showed ataxia with brisk reflexes in both upper and lower limbs. MRI brain showed diffuse cerebral and cerebellar atrophy with normal thalamus (**Figure 1B**). Electroencephalogram revealed a normal background and generalised spikes, polyspike and wave activity.

P3 is an 8 year old female child who first presented at the age of 5 years with visual failure in both eyes. Examination showed visual acuity of counting fingers close to face in the right eye (RE) and 6/90 on Snellen's chart in the left eye (LE). Fundus showed bilateral optic disc pallor with severe arteriolar attenuation and diffuse pigment mottling. SD-OCT showed severe loss of photoreceptors and foveal thinning. A full field ERG showed severe rod-cone dysfunction. The child developed seizures at the age of 6 years. MRI brain showed mild cerebral atrophy, normal cerebellum and thalamus. On last examination, vision was PL in both eyes, her seizures were under control with medications. The unaffected sibling (C3) and his parents (C1 and C2) had normal vision, normal fundus examination and no seizures.

### Exome capture and high-throughput sequencing

DNA from the parents and three of the children (P2, P3 and C3) was extracted from blood lymphocytes, using standard procedures. DNA samples could not be obtained from P1. The P2, P3 and C3 samples were subjected to whole-exome sequencing. This involved 3.5 micrograms of each sample being made up to 100 ul with TE and sonicated to fragment DNA into sizes ranging from 100 bp to 200 bp (Covaris). Such fragmented DNA was cleaned up using Agencourt AMPure XP beads (Beckman Coulter). Size distribution was checked by running an aliquot of the sample on an Agilent High Sensitivity Bioanalyzer chip. Genomic DNA libraries were then constructed using Ion TargetSeq Exome Enrichment for the Ion Proton System (Part # MAN0006730, revision 5.0). End-repair and adapter ligation were done according to established protocol (Ion plus fragment library kit # 4471252). The samples were cleaned using Ampure XP beads. The samples were size-selected on gel at 200 bp and eluted using MinElute columns. Adaptor-ligated fragments were enriched by PCR amplification. The prepared libraries were pooled in equal amounts (167 ng each) and concentrated using a vacuum concentrator to give ∼500 ng. The library fragments were captured in solution using ∼2 million TargetSeq capture probes, producing biotinylated oligos ranging in size from ∼50 bases to ∼120 bases (47°C for 66 hours). Hybridisation specificity was ensured by using blocker DNA sequences (Human Cot-1 DNAR Fluor QC and Ion TargetSeq Blockers). Bound DNA was isolated using M 270 streptavidin-coated DynabeadsR paramagnetic beads and then amplified and purified. An aliquot of the captured library was run on an Agilent High Sensitivity Bioanalyzer Chip. Real time PCR validation involved using pre- and post-capture libraries for observing capture efficiency. The purified, exome-enriched library was then used to prepare a template on particles for preparing enriched, template-positive Ion PI ion sphere particles (ISPs) for sequencing on an Ion PI chip to obtain the necessary data coverage.

### Reference genome and RefSeq database

The human reference genome GRCh37/hg19 was used for mapping exome-sequencing (Exome-Seq). The sequence database downloaded from the Ion Torrent reference website (http://updates.iontorrent.com/reference/hg19.zip) was used as our gene model and for determining amino acid substitutions.

### Exome-Seq processing - Read mapping, variant calling and effect determination

Raw data (which is available upon request) was obtained from the Ion Proton sequencer and Ion Suite was used for checking data quality and filtering. The high quality filtered data was aligned with the human reference genome and variants were named using Ion Reporter v1.6 (Ion Suite). Variations (vcf file) were then transferred to a local Linux machine. The filtered variations were annotated, based on their function (e.g. synonymous, non-synonymous) and their potential effect by using Ingenuity software plugin within the Ion Reporter. The results were further compared with the dbSNP database NCBI version db 137. This result helped to identify novel variations and identify their effect. Finally, the annotated variations were further reviewed manually. Genotypic Technology (Bangalore, Karnataka, India) did the exome sequencing and primary data analysis. The complete filtered data has been included as **Tables S1, S2 and S3**.

### *MFSD8* direct sequencing and *in silico* analysis

The *MFSD8* exon 12 encoding sequence (ENST00000296468) was amplified by PCR in all available DNA samples from the family members using exon-flanking oligonucleotides. Amplicons were purified using shrimp alkaline phosphatase and exonuclease I, and sequenced with internal primers using Sanger sequencing method (primer sequences and PCR conditions are available on request). ClustalW software was used for aligning the human MFSD8 wild type sequence (Uniprot ID: Q8NHS3) with those from the following vertebrate species: *Pan troglodytes* (H2QQ56), *Mus musculus* (Q8BH31), *Bos taurus* (E1BPY5), *Xenopus laevis* (Q6GPQ3) and *Danio rerio* (Q0VA82). SIFT (http://sift.bii.a-star.edu.sg/) and PolyPhen2 (http://genetics.bwh.harvard.edu/pph2/) software was used for ascertaining potential deleterious effects caused by the p.Trp407Arg and p.Met454Thr mutations. Briefly, PolyPhen2 and SIFT prediction values resulted from algorithms which included protein characteristics such as comparative analysis of sequences from different species and exchanged amino acids' physicochemical characteristics. SIFT values lower than 0.06 were considered potentially pathogenic. PolyPhen2 results were assessed as being probably damaging (more confident prediction), possibly damaging (less confident prediction) or benign (non-pathogenic). The Amino Acid Mutation Stability Prediction Server MUpro (version 1.0, http://mupro.proteomics.ics.uci.edu/) was used to study potential MFSD8 stability impairment due to the p.Trp407Arg and p.Met454Thr mutations [11].

## Results

### Whole-exome sequencing

Whole-exome sequencing produced ∼3.4GB data for 3 samples for each individual as single-end, having 113 bp mean read length and about 76% (50 Mb in length) of the targeted bases were covered at ∼44 average read depth which sufficiently passed our thresholds for calling SNPs and short insertions or deletions (indels). Bases having quality scores above 20 (99% accuracy regarding a base call) represented over 80%–82% of total sequence data. NGS data filtering revealed that P2 and P4 shared 2 homozygous missense variants located on the *MFSD8* coding region: c.1219T>C (p.Trp407Arg) and c.1361T>C (p.Met454Thr). NGS showed that C3 was a heterozygous carrier for the p.Trp407Arg and p.Met454Thr mutations. These variants were not present in SNP public databases (e.g. dbSNP database NCBI version db 137).

### Direct sequencing and *in silico* analysis of the *MFSD8* p.Trp407Arg and p.Met454Thr mutations

Direct sequencing confirmed the aforementioned NGS results and revealed that P2 and P4 had inherited mutant alleles from their parents. Multiple protein alignment revealed a strict conservation, during vertebrate species' evolution, of both tryptophan and methionine residues at 407 and 454 positions. SIFT was 0.00 and Polyphen2 1.00 (“probably damaging”) for both mutations. Analysis of amino acid mutation stability for p.Trp407Arg and p.Met454Thr mutations identified in this study using MUpro software suggested a decrease in the stability of *MFSD8* protein structure.

## Discussion

**Figure 2:**
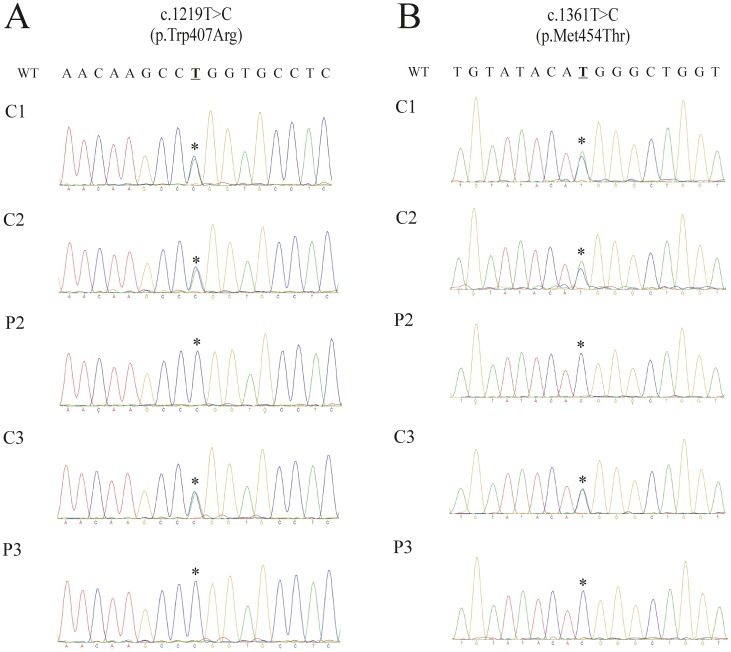
Chromatograms showing *MFSD8* c.1219T>C (A) and c.1361T>C (B) mutations. WT: wild type sequence. C1: control 1 (III:6). C2: control 2 (III:7). P2: patient 2 (IV:2). C3: control 3 (IV:3). P3: patient (IV:4). Asterisks show the accurate position of relevant mutations.

**Figure 3:**
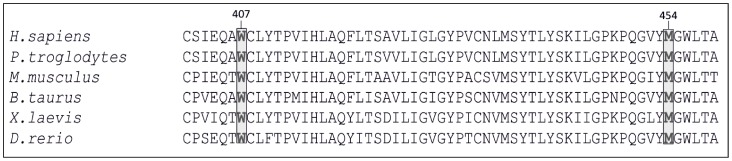
Comparative sequence analysis of MFSD8 orthologues. Residues at position 407 (W) and 454 (M) are indicated in bold and highlighted with grey shading.

A rapid clinical diagnosis of NCL may be particularly challenging since clinical signs may be unspecific, similar to many other neurodegenerative disorders. Electron microscopy, showing ultrastructural lipofuscinic pigments, has been used for diagnosing NCL. *CLN* genotyping has led to establishing molecular diagnosis which may be related to specific clinical subtypes. However, mutations in distinct *CLN* genes may cause similar phenotypes, thereby hampering candidate gene selection for direct sequencing. The patients in the present study were affected by a recessive neurodegenerative disease, mainly characterised by progressive visual dysfunction, epilepsy and gradual regression of milestones. Such features, as well as a family history of consanguinity, were clinically evocative of a recessive late infantile onset form of ceroid lipofuscinosis. However, a single candidate gene could not be selected for direct sequencing, since CLN2, CLN5, CLN6, CLN7 and CLN8 patients may share clinical features to those presented by our patients. This would have meant that definitive molecular diagnosis by direct sequencing would have been time-consuming and expensive. Our group, and others, have previously shown that whole-exome sequencing is a cost-effective approach to establishing a molecular diagnosis of Mendelian recessive diseases, particularly when mutations in distinct genes are related to overlapping phenotypes [12], [13]. Therefore, for rapid determination of the molecular aetiology we performed whole-exome sequencing for two of the affected children (P2 and P4) and their unaffected brother (C3) (**Figure 1**). Since the family tree revealed consanguinity, it was estimated that the most likely genetic hypothesis was related to a homozygous non-synonymous pathogenic mutation in the patients. NGS data analysis revealed that P2 and P3 shared a total of 2 novel *MFSD8* homozygous coding variants (c.1219T>C, p.Trp407Arg and c.1361T>C, p.Met454Thr) (**Figure 2**). Direct sequencing confirmed that the patients' relatives (C1, C2 and C3) carried both variants at heterozygous state. *In silico* analysis of the p.Trp407Arg and p.Met454Thr mutation revealed that the W and M residues (in 407 and 454 positions) had been strictly conserved during vertebrate species evolution, thereby underlining its critical functional role (**Figure 3**). Furthermore, these mutations implied drastic modification in terms of physicochemical properties. For instance, the W is a hydrophobic aromatic amino acid while R is a positively-charged residue. Accordingly, SIFT and Polyphen bioinformatics tools predicted a deleterious effect for both substitutions as they determined complete intolerance for the arginine and threonine residue at positions 407 and 454, respectively. Furthermore the MuPro bioinformatics tool predicted MFSD8 protein instability due to the p.Trp407Arg and p.Met454Thr mutations. These findings, as well as the lack of theses variants in public SNP databases, led to functionally linking them to the patients' phenotype. The p.Trp407Arg mutation was located in a highly conserved region of amino acids between the MFSD8 protein's 9^th^ and 10^th^ transmembrane domains, while the p.Met454Thr mutation is located on the 11^th^ transmembrane domain. This might have been related to correct polypeptide folding and protein's normal function. It is important to note that these two novel variations were located in the same disease allele because the unaffected patients' parents and C3 were carriers of both mutations at heterozygous state.

Taken together, our results demonstrate that whole-exome sequencing is a powerful diagnostic tool for enabling the rapid determination of variant late-infantile neuronal ceroid lipofuscinosis molecular aetiology. We estimate that this method might be commonly used to determine the molecular basis of NCL. We also provide clinical information regarding this type of patient which might be useful for future genotype-phenotype correlations. We hope that these findings will contribute to genetic counseling especially for consanguineous families with NCL. Finally, functional tests are necessary to establish the accurate pathogenic effect of mutations described in the present study.

## Supporting Information

Table S1 **Filtered exome sequencing data from Patient 2.**
(XLS)

Table S2 **Filtered exome sequencing data from Control 3.**
(XLS)

Table S3 **Filtered exome sequencing data from Patient 3.**
(XLS)
